# Improving Care for Persons With Cognitive Impairment in Community-Based Long-Term Care and Acute Care Settings

**DOI:** 10.1093/geroni/igab046.251

**Published:** 2021-12-17

**Authors:** Jing Wang, Bei Wu

## Abstract

The COVID-19 pandemic has highlighted the importance of providing person-centered care for our vulnerable populations across the globe. This symposium focuses on improving care for persons with cognitive impairment and dementia in various care settings. The first study explored dyadic experiences of living with early-onset dementia pre and during COVID-19 in China through a person-centered care lens. The second concept analysis presented four interrelated dimensions of Asian American caregiver support, including individual, family, community, and professional health system. The third study investigated undergraduate nursing students’ attitudes toward pursuing jobs of providing care for older adults with dementia in rapidly-aging urban areas in China and its associated factors. The fourth study examined the impact of social isolation on cognitive function and Quality of Life among acute ischemic stroke (AIS) patients in China. The last study explored an association between perceived control and cognitive function among acute ischemic stroke (AIS) patients in China. The last two studies suggested that perceived control and social isolation may be potential targets in cognitive interventions for AIS patients. This symposium presents the understanding of dementia caregiver support, the empirical evidence of living with dementia, the attitudes towards dementia care among the next generation of nursing workforce, and the impact of social factors on cognitive functions longitudinally. The presenters emphasize the importance of improving care in long-term care and acute care settings. There is a need to design person and family-centered care to improve health and wellbeing of persons with cognitive impairment.

